# IL-17A, IL-17RC polymorphisms and IL17 plasma levels in Tunisian patients with rheumatoid arthritis

**DOI:** 10.1371/journal.pone.0194883

**Published:** 2018-03-27

**Authors:** Tarak Dhaouadi, Mayssa Chahbi, Youssra Haouami, Imen Sfar, Leila Abdelmoula, Taieb Ben Abdallah, Yousr Gorgi

**Affiliations:** 1 Research Laboratory in Immunology of Renal Transplantation and Immunopathology (LR03SP01), Charles Nicolle Hospital, Tunis, Tunisia; 2 Department of Rheumatology, Charles Nicolle Hospital, Tunis, Tunisia; Peking University First Hospital, CHINA

## Abstract

**Background:**

Interleukin-17 (IL-17), a cytokine mainly secreted by Th17 cells, seems to play a significant role in the pathogenesis of rheumatoid arthritis (RA). Functional genetic polymorphisms in IL-17 and its receptor genes can influence either qualitatively or quantitatively their functions. Therefore, we aimed to study the impact of IL17-A and IL17RC polymorphisms on plasma level of IL-17 and RA susceptibility and severity.

**Methods:**

In this context, IL-17A*rs2275913 and IL-17RC*rs708567 polymorphisms were investigated together with the quantification of IL17 plasma level in 115 RA patients and 91 healthy control subjects matched in age, sex and ethnic origin.

**Results:**

There were no statistically significant associations between IL-17A and IL-17RC studied polymorphisms and RA susceptibility. In contrast, IL-17A plasma levels were significantly higher in patients (55.07 pg/ml) comparatively to controls (4.75 pg/ml), *p*<10E-12. A ROC curve was used to evaluate the performance of plasma IL-17 in detecting RA. Given 100% specificity, the highest sensitivity of plasma IL-17A was 61.7% at a cut-off value of 18.25 pg/ml; *p* < 10E-21, CI = [0.849–0.939]. Analytic results showed that the IgM-rheumatoid factor and anti-CCP antibodies were significantly less frequent in patients with the IL-17RC*A/A genotype than those carrying *G/G and *G/A genotypes; *p* = 0.013 and *p* = 0.015, respectively. Otherwise, IL-17 plasma levels’ analysis showed a significant association with the activity of RA (DAS28≥5.1 = 74.71 pg/ml vs. DAS28<5.1 = 11.96 pg/ml), *p*<10E-6.

**Conclusion:**

IL-17A*rs2275913 (G/A) and IL-17RC*rs708567 (G/A) polymorphisms did not seem to influence RA susceptibility in Tunisian population. This result agrees with those reported previously. Plasma IL-17A level seems to be predictive of severe RA occurrence.

## Introduction

Rheumatoid arthritis (RA) is a systemic autoimmune disease which is characterized by symmetric inflammation in synovial tissue. This synovitis leads to both joint cartilage and bone destruction. In spite of recent advances in the understanding of the immuno-pathophysiology of RA, the puzzle is still incomplete. The predominance of activated T-cells infiltrates in the synovium is the hallmark of RA [1]. Classically, in RA, the key player is the imbalance in Th1/Th2 subsets of T helper cells which is characterized by an enhanced Th1 activation that plays a critical role in the disease induction [2]. However, the Th1/Th2 imbalance failed to explain the absence of IFN-γ in RA synovium and the lack of efficacy of anti-IFN-γ biotherapies [3]. Moreover, in recent years the classical Th1/Th2 paradigm view was challenged by the discovery of Th17 and Treg subsets which are marked by opposite effects on autoimmune diseases.

Th17 cells were found to be crucial for host defense responses by releasing their effector cytokines, IL-17A (also called IL-17) and IL-17F. In fact, deficiency of IL-17A and IL-17F in mice caused increased susceptibility to the infection of extracellular pathogens, such as *Klebsiella pneumonia*, *Citrobacter rodentium*, and *staphylococcus aureus* [4, 5].

Since the identification of the Th17 lineage, many studies have focused on Th17 cells role in both murine models and human counterparts of autoimmune diseases. Indeed, Th17 were found to be the central effector lineage in experimental autoimmune encephalomyelitis [6], a murine model for multiple sclerosis (MS). Moreover, the expression of IL-17A and its producing T cells were highly elevated in brain lesion areas of MS patients [7]. Likewise, the understanding of the pathophysiology of psoriasis has shifted from the Th1 to the Th17 perspective [8] as well as in inflammatory bowel diseases [9] and systemic lupus erythematosus [10]. Much like in these autoimmune diseases, the potential role of Th17 has been largely investigated in RA. In fact, in experimental murine models of RA, blockade of IL-17 improved [11], while its induced overexpression worsened diseases’ evolution [12]. Furthermore, IL-17A-deficient mice were protected from either collagen induced arthritis or spontaneous autoimmune arthritis [5, 13]. Moreover, and in human, a recent meta-analysis [14] confirmed the effectiveness of the anti-IL17 therapy in improving RA patients even if it suffers from short duration of included studies.

IL-17A can form homodimers, or heterodimers with IL-17F, even if the IL-17A homodimer is significantly more powerful [15]. IL-17A/F signalling passes through a dimeric receptor, IL-17RA/IL-17RC which has a unique structure architecture combining 2 fibronectin III-like domains in its extracellular region and a SEF/IL-17R (SEFIR) intracellular domain [16]. Both IL-17A/F and their receptors genes exhibit functional polymorphisms that may alter qualitatively and/or quantitatively their expressions and hence influence predisposition to autoimmune diseases. In fact, many studies have investigated the potential role of both IL-17 gene polymorphisms and IL-17 levels in RA susceptibility [17]. Actually, in the meta-analysis of Lee et al [16], IL-17A rs2275913 and IL-17F rs763780 polymorphisms were associated to RA in Caucasians, while the IL-17A rs8193036 was not.

Thus, the aim of the present study was to investigate the impact of IL-17A rs2275913, IL-17RC rs708567 genes polymorphisms on IL-17A plasma levels together with their influence on RA susceptibility as well as its severity.

## Material and methods

### Subjects

This study included 115 RA patients and 91 healthy voluntary blood donors from the same ethnic origin (Tunisian). Patients were consulting at the rheumatology department of the Charles Nicolle Hospital in Tunis and were diagnosed according to the “2010 Rheumatoid Arthritis Classification Criteria” [18]. Clinical and biological features of patients are recorded in Table 1.

**Table 1:** Clinical and biological features of RA patients.

Patients	n = 115	Controls n = 91	*p*
**Sex ratio (Men / Women)**	0.22 (21/94)	0.26 (19/72)	0.63
**Mean age ± DS (years)**	50.77 ± 13.6	48,48 ± 9,2	0,173
**Mean onset age ± DS (years)**	41.38 ± 14.6	_	_
**Median of disease’s duration [1**^**st**^**-3**^**rd**^ **quartiles] (months)**	85 [43–185.25]	_	_
**Mean DAS28 ± DS**	5.907 ± 1.675	_	_
**Active disease (DAS28 > 5 .1)**	79 (68.7%)	_	_
**Bone lesions n (%)**	55 (48.7%)	_	_
Bone erosions n (%)	32 (27.8%)	_	_
Osteoporosis n (%)	23 (20%)	_	_
**Subcutaneous nodules n (%)**	23 (20%)	_	_
**Anti-CCP antibodies (ACCPA) + n (%)**	81 (70.4%)	_	_
**Mean level of ACCPA ± DS (RU/ml)**	61.08 ± 56.81	_	_
**Rheumatoid factor (RF) + n (%)**^a^	78 (67.8%)	_	_
**RF level > 100 IU/ml n (%)**	63 (54.8%)	_	_
**IgM-RF + n (%)**^b^	71 (61.7%)	_	_
**Mean level of IgM-RF (IU/ml)**	165.94	_	_
**IgA-RF + n (%)**^c^	61 (53%)	_	_
**Mean level of IgA-RF (IU/ml)**	204.17	_	_

^a^performed in only 103 patients

^b^performed in only 103 patients

^c^performed in only 103 patients

Controls were healthy subjects matched in age, gender and ethnicity. Ethnicity (Tunisian) of both patients and controls was determined by an oral questionnaire. None of the healthy subjects had any evidence of personal or family history of RA or any autoimmune disease.

All patients and controls gave written informed consent to participate in the study, and the local Ethics’ committee of Charles Nicolle Hospital approved this study. No benefits in any form have been received or will be received from a commercial party related directly or indirectly to the subject of this manuscript.

### Blood sampling and genotyping

Genomic DNA was isolated from EDTA peripheral blood samples of all patients and extracted by standard salting-out procedure [19]. The identification of the IL-17A rs2275913 (A/G -197) and the IL-17RC rs708567 (G/A +6313) polymorphisms were performed by *polymerase chain reaction restriction fragment length polymorphism* (PCR-RFLP) using specific primers: [(IL-17A*F: 5’-AACAAGTAAGAATGAAAAGAGGACATGGT-3’; IL-17A*R: 5’-CCCCCAATGAGGTCATAGAAGAATC-3’) and (IL-17RC*F: 5’-AGTAGGGTAGGCCTGGAAGG-3’; IL-17RC*R: 5’- CACTGGGAAGAGCCTGAAGA-3’), respectively (metabion® international AG, Lena-Christ-strasse 44I, D-82152 Martinsried, Deutshland)] followed by a digestion of the amplification products using EcoNI and HinfI enzymes respectively.

### Plasma IL-17A quantification

Plasma concentrations of IL-17A were measured using a home-made sandwich ELISA with reagents for human IL-17 detection (human IL-17 capture antibody, human IL-17 detection antibody, human IL-17 standard, and streptavidin-HRP) from R&D SYSTEMS^TM^ manufacturer. Additional materials and solutions including 96-well microplate, plate sealers, phosphate-buffered saline (PBS), wash buffer, reagent diluent, substrate solution and stop solution were also used to perform IL-17 quantification according to manufacturer’s recommendations.

### Statistical analysis

Results of continuous quantitative variables (Age, onset age, evolution period, DAS28, anti-CCP antibodies, rheumatoid factors and IL-17 plasma levels) are expressed as means ± SD, and means of groups were compared by ANOVA-test (SPSS 11 Inc. Chicago, Illinois, USA). Receiver-operating characteristic (ROC) curves were used to assess the plasma IL-17 performance in RA diagnosis and activity.

For qualitative variables, univariable analysis was performed using chi-square test or Fisher’s exact test for small numbers (SPSS 11 Inc. Chicago, Illinois, USA). Values of *p* < 0.05 were considered to be statistically significant. In order to evaluate the strength of associations, odds ratios (OR) together with 95% confidence intervals (CI) were calculated. Logistic regression models were built according to age and gender to estimate adjusted ORs. Chi-square test for trend was used to evaluate the association between a nominal variable (active RA) and an ordinal variable (genotypes).

## Results

In total, 115 RA patients and 91 healthy control subjects were included. Mean age of the studied group was at 50.77 ± 13.6 years with a sex ratio (Men/Women) of 0.22 (21/94). Mean onset-age was at 41.38 ± 14.6 years with a median of disease’s duration of 85 [43–185.25] months. Mean DAS28 was at 5.907 ± 1.675 with 79 (68.7%) patients with active disease (DAS28 > 5.1). Bone lesions were found in 55 (48.7%) patients while subcutaneous nodules were noted in only 23 (20%) patients. Serologically, anti-CCP antibodies (ACCPA) and rheumatoid factors (RF) were present in 81 (70.4%) and 78 (67.8%), respectively (Table 1).

### Plasma IL-17 levels in patients and controls

**Fig 1:**
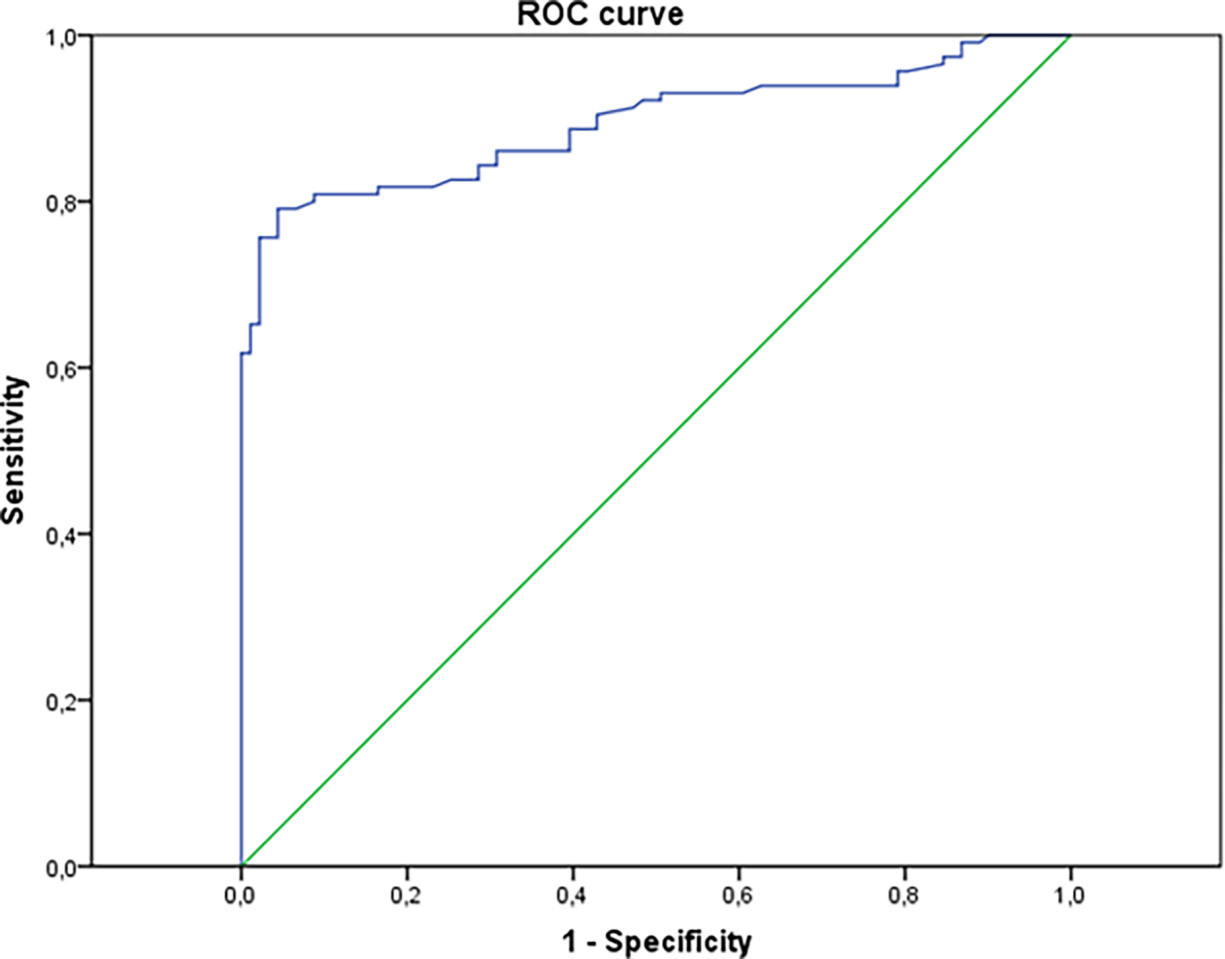
ROC curve study to evaluate the performance of plasma IL-17 concentration in detecting RA. The area under the ROC curve is 0.894; *p* < 10E-21, CI 95% = 0.849–0.939. For a 100% specificity, sensitivity was about 61.7% at a cutoff value of 18.25 pg/ml.

Plasma IL-17 concentration was significantly higher in RA patients (55.07 pg/ml) comparatively to controls (4.75 pg/ml), *p* < 10E-12. In order to determine the ability of plasma IL-17 in identifying RA, a ROC curve was used (Fig 1). For a 100% specificity, the maximum of sensitivity was about 61.7% at a cut-off value of 18.25 pg/ml with an area under the curve of 89.4%; *p* < 10E-21, CI 95% = 0.849–0.939.

**Fig 2:**
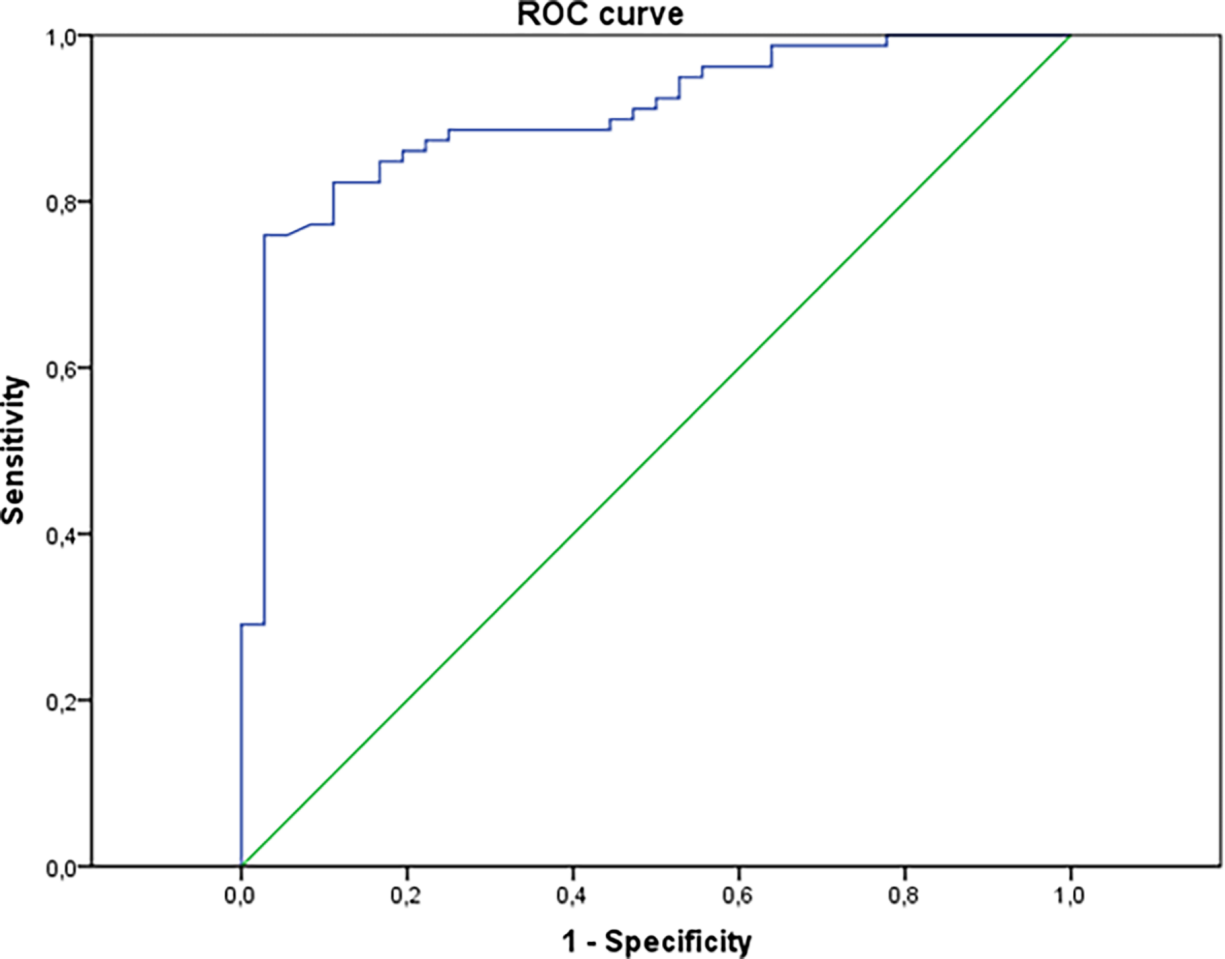
ROC curve to assess the ability of plasma IL-17 level in discerning between active and non-active RA. The area under the ROC curve is 0.903; *p* < 10E-11, CI 95% = 0.846–0.961. For a specificity of 97.2%, the sensitivity was about 75.9% at a threshold of 26.55 pg/ml.

Moreover, plasma IL-17 levels were significantly higher in active disease (DAS28>5.1), 74.71 vs 11.06 pg/ml, *p* < 10E-6. Furthermore, there was a significant correlation between IL-17A levels and DAS28 values, Spearman *r* = 0.558, *p* < 10E-10. Again, a ROC curve was used to determine the performance of plasma IL-17 in detecting active disease. The area below the curve was 0.903; *p* < 10E-11, CI 95% = 0.846–0.961 (Fig 2). Thus, with a specificity of 97.2% the sensitivity was about 75.9% at a threshold of 26.55 pg/ml.

Inversely, there were no significant association between IL-17 and the other clinical and biological features of RA such as gender, subcutaneous nodules, bone lesions and both positivity and levels of ACCPA and RF.

### Analysis of IL-17A rs2275913 polymorphism

There were no significant differences in genotypes and alleles frequencies between patients and controls (Table 2).

**Table 2:** Results of IL-17A rs2275913 and IL-17RC rs708567 genotyping in patients and controls.

IL-17A Genotype	Patients n = 115	Controls n = 91	*p*	OR (95% CI)
**G/G**	11 (9.6%)	9 (9.8%)	0.92	
**G/A**	86 (74.8%)	66 (72.5%)		_
**A/A**	18 (15.6%)	16 (17.6%)		
**IL-17A Allele**				
**G**	0.469	0.461	0.55	1.13 [0.74–1.7]
**A**	0.531	0.549		
**IL-17RC Genotype**				
**G/G**	48 (41.73%)	34 (37 .36%)	0.44	
**G/A**	48 (41.73%)	46 (50 .54%)		_
**A/A**	19 (16.52%)	11 (12.08%)		
**IL-17RC Allele**				
**G**	0.626	0.626	0.96	1.01 [0.66–1.55]
**A**	0.374	0.374		

Analytic results showed that, even lacking significance, there was a trend of associations between subcutaneous nodules and the IL-17A*A/A genotype; and osteoporosis with the IL-17A*G/G genotype (Table 3). Otherwise, the IL-17A studied polymorphism was not associated to the activity of the RA in our patients. Likewise, there was no correlation between the IL-17A polymorphism with either plasma autoantibodies (ACCPA and RF) or plasma IL-17A concentration (Table 3).

**Table 3:** Analytic results of the IL-17A rs2275913 polymorphism.

Genotype	G/G n = 11	G/A n = 86	A/A n = 18	*p*	OR [95% CI]
**Mean onset age**	39.27 ± 16.24	42.72 ± 14.11	36.44 ± 15.32	0.225	_
**Mean DAS28**	5.39 ± 1.37	5.87 ± 1.72	6.04 ± 1.66	0.923	_
**DAS28 > 5,1 n (%)**	7 (63.6%)	57 (66.3%)	15 (83.3%)	0.34	_
**Subcutaneous nodules n(%)**	1 (9.1%)	15 (17.6%)	7 (38.9%)^a^	**0.029**	**3.22 [0.95–10.86]**
**Bone lesion n (%)**	6 (54.5%)	40 (46.5%)	10 (55.6%)	0.721	_
Bone erosions n (%)	1 (9.1%)	27 (31.4%)	5 (27.8%)	0.13	_
Osteoporosis n (%)	5 (45.5%)^b^	13 (15.1%)	5 (27.8%)	**0.041**	**3.98 [0.92–17.07]**
**ACCPA + n (%)**	9 (81.8%)	61 (70.9%)	11 (61.1%)	0.485	_
**Mean ACCPA level RU/ml**	81.58	58.35	61.61	0.446	_
**RF + n (%)**	9 (81.8%)	56 (75.7%)	13 (72.2%)	0.843	_
**RF > 100 IU/ml n (%)**	8 (72.7%)	44 (60.3%)	11 (61.1%)	0.931	_
**IgM-RF + n (%)**	7 (63.6%)	51 (68.9%)	13 (72.2%)	0.889	_
**Mean IgM-RF level IU/ml**	150.91	114	165.94	0.482	_
**IgA-RF + n (%)**	8 (72.7%)	44 (59.5%)	9 (50%)	0.48	_
**Mean IgM-RF level IU/ml**	314.55	190.54	192.78	0.283	_
**Plasma IL-17A level pg/ml**	59.18	52.3	65.8	0.705	_

^a^ Chosen IL-17A genotype compared with the 2 other genotypes

^b^Chosen IL-17A genotype compared with the 2 other genotypes

### Analysis of IL-17RC rs708567 polymorphism

Genotypes and alleles distributions of the IL-17RC polymorphism were quite similar between RA patients and controls (Table 2).

Analytic study revealed that there was a trend of increase in the frequency of active disease in patients with IL-17RC*A/A genotype (84.2%) comparatively to those carrying *G/G (58.3%) and *G/A (72.9%) genotypes, *p* = 0.085. In addition, trend analysis showed the frequency of active RA significantly increased with the acquisition of the IL-17RC*G allele, χ^2^_trend_ = 4.91, *p* < 0.05 (Table 4). Besides, the frequency of plasma ACCPA was significantly higher in patients with IL-17RC*G/G (79.2%) and *G/A (70.8%) genotypes than in the case of *A/A genotype (47.4%); *p* = 0.015, OR [95% CI] = 0.3 [0.1–0.92] (Table 4). Likewise, IgM-RF autoantibodies were more frequent in patients with IL-17RC*G/G (33/45; 73,3%) and *G/A (30/40; 75%) genotypes than in those with *A/A genotype (8/18; 44.4%); *p* = 0.013, OR [95% CI] = 0.28 [0.1–0.8] (Table 4). Inversely, the IL-17RC polymorphism was not associated with the presence of either subcutaneous nodules or bone lesions (Table 4). Likewise, there were no significant differences in the plasma IL-17A levels between the three IL-17RC genotypes (Table 4).

**Table 4:** Analytic results of the IL-17RC rs708567 polymorphism.

Genotype	G/G n = 48	G/A n = 48	A/A n = 19	*p*	OR [95% CI]
**Mean onset age**	40 ± 16.19	42.15 ± 13,46	43 ± 13.36	0.686	_
**Mean DAS28**	5.63 ± 1.66	5.98 ± 1.56	6.41 ± 1.91	0.21	_
**DAS28 > 5,1 n (%)**	28 (58.3%)	35 (72.9%)	16 (84.2%)	<0.05^a^	_
**Subcutaneous nodules n(%)**	8 (16.7%)	11 (23.4%)	4 (21.1%)	0.712	_
**Bone lesion n (%)**	25 (52.1%)	21 (43.8%)	9 (50%)	0.707	_
Bone erosions n (%)	16 (33.3%)	13 (27.1%)	3 (16.7%)	0.2	_
Osteoporosis n (%)	9 (18.8%)	8 (16.7%)	6 (33.3%)	0.16	_
**ACCPA + n (%)**	38 (79.2%)	34 (70.8%)	9 (47.4%)^b^	**0.015**	**0.3 [0.1–0.92]**
**Mean ACCPA level RU/ml**	65.59 ± 53.36	62.76 ± 60.33	45.44 ± 56.4	0.413	_
**RF + n (%)**	36 (80%)	31 (77.5%)	11 (61.1%)	0.272	_
**RF > 100 IU/ml n (%)**	31 (68.9%)	23 (57.5%)	9 (52.9%)	0.267	_
**IgM-RF + n (%)**	33/45 (73.3%)	30/40 (75%)	8/18 (44.4%)^c^	**0.013**	**0.28 [0.1–0.8]**
**Mean IgM-RF level IU/ml**	199.22	149.05	120.28	0.342	_
**IgA-RF + n (%)**	28 (62.2%)	22 (55%)	11 (61.1%)	0.783	_
**Mean IgM-RF level IU/ml**	239.04	163.07	208.33	0.347	_
**Plasma IL-17A level pg/ml**	45.46	59.6	67.88	0.357	_

^a^ χ^2^_trend_ = 4.91, *p* < 0.05

^b^Chosen genotype compared with the 2 other genotypes

^c^Chosen genotype compared with the 2 other genotypes

## Discussion

Recent studies have shown that Th17 cells and their effector cytokines, IL-17A and IL-17F, play a significant role in RA susceptibility as well as in its severity and the response to treatment [20, 21]. Thus, we examined the impact of plasma IL-17 level on both RA predisposition and disease activity. Yet, the production of this cytokine is genetically determined and probably controlled by epigenetic factors. Consequently, we investigated polymorphisms in IL-17A and its receptor IL-17RC genes in Tunisian RA patients and evaluated their association with disease severity and plasma IL-17 concentration.

In the present study, we noted that plasma IL-17A levels were significantly higher in RA patients than in healthy subjects, *p* < 10E-12. This result corroborates those found in Serbian [22], Taiwanese [23], British [24], Chinese [25] and Tunisian [26] populations. Moreover, the meta-analysis of Lee YH et al [17] definitely confirmed this association between RA and raised levels of circulating IL-17. Using a ROC curve, we determined a cut-off level of 18.25 pg/ml that confers to plasma IL-17 a sensitivity of 61.7% with a 100% specificity. This finding was nearly the same in another study made in 108 Tunisian RA patients and 202 healthy controls [26], in which for a cut-off value of 23 pg/ml of serum IL-17, the sensitivity and the specificity were 55.56% and 100% respectively. Nevertheless, as elevated levels of IL-17 were also noted in other autoimmune diseases [7–10], this cytokine cannot be used as a biomarker for RA diagnosis. In addition, we noted a significant correlation between plasma IL-17 concentration and the RA activity, *r* = 0.558, *p* < 10E-10. Likewise, the IL-17 circulating levels were associated to the disease activity score (DAS28) in other published studies [21, 26], suggesting that IL-17 could be used to assess the disease outcome. Furthermore, in a study performed 48 Taiwanese RA patients [23], the mean level of IL-17 decreased significantly after anti-TNF treatment in responders, while it significantly increased in non-responders. This result highlights the usefulness of circulating IL-17 in defining the activity of RA and in predicting the outcome under therapy.

In this study, no significant association was found for IL-17A rs2275913 and disease susceptibility, *p* = 0.92. This result confirmed a previous report in the Tunisian population [26]. Again, most of published studies performed in Algerian [27], Polish [28, 29], Brazilian [30] and Chinese [31] populations emphasized the lack of association between the IL-17A rs2275913 and RA risk. Nevertheless, two studies performed in Norwegian [32] and Brazilian [33] reported that the rs2275913 polymorphism increased risk of RA. In Norwegian patients, the IL-17A*G allele conferred a weak risk, OR = 1.17, 95% CI = 1.02–1.34 [32]. While the risk for RA was moderate for the IL-17*G/G genotype, OR = 3.18, 95% CI = 1.13–9.95 in the Brazilian study [33]. Accordingly, the meta-analysis of Lee et al [17] the IL-17A*A allele conferred a weak protective role for RA risk, OR = 0.866; 95% CI = 0.794–0.944. It is of note, that in this meta-analysis the highest weight of the included studies was for the Norwegian report [32] as it involved 950 RA patients and 933 healthy controls. Therefore, the risk conferred by IL-17A rs2275913 G allele in RA predisposition might be weak and that could explain the absence of association reported in the mainstream of published studies as well as the present study. Besides, we did not note any association for the IL-17A polymorphism and RA activity. This data corroborates results of previous reports in diverse populations. However, Ouled Salah et al [26] reported that patients with one copy of IL-17A*A allele were good responders to methotrexate therapy. Likewise, the IL-17*G/G genotype was predictive for highest activity in patients under anti-TNF therapy in a Polish study [28]. The rs2275913 polymorphism in the IL-17A gene is located in the promoter at position -197. Till now its functional impact is unknown, but the current data suggest that it may enhance the promotor activity that would results in a higher cytokine secretion.

In this study, we reported no significant association for the IL-17RC rs708567 polymorphism with RA risk. To our knowledge this is the first study to analyse its impact on RA susceptibility. The rs708567 polymorphism is located in the exon 4 of the IL-17RC gene (G/A +6313), but its functional effect is indefinite. It was reported that the IL-17RC*G/G genotype was significantly associated to the curve severity of adolescent idiopathic scoliosis [34]. Whereas this genotype conferred a lower risk for severe malaria and patients had the lowest parasite burden [35]. Therefore, the IL-17RC*G allele could possibly confer a better affinity to IL-17A and IL-17F. Likewise, we showed that IL-17RC*G allele was associated to the presence of anti-CCP and IgM-RF autoantibodies. Inversely, this study revealed a significant trend of raise of DAS28 with IL-17RC*A allele. Nevertheless, these results have to be confirmed with other studies in independent cohorts.

## Conclusions

The IL-17A*rs2275913 (G/A) and IL-17RC*rs708567 (G/A) polymorphisms did not seem to influence RA susceptibility in Tunisian, but may influence its severity. Plasma IL-17A seems to be predictive of severe RA occurrence.

## Supporting information

S1 File Patient consent form. This document describes the consent that was signed by each patient included in this study.
(PDF)

S2 File Database of RA patients. This file depicts the data acquired from the cohort of patients with rheumatoid arthritis.
(XLSX)

S3 File Database of controls. This file shows the controls features.
(XLSX)
