# Supplementary material for: IL-17A, IL-17RC polymorphisms and IL17 plasma levels in Tunisian patients with rheumatoid arthritis
Source: PLoS One. 2018 Mar 27;13(3):e0194883. doi: 10.1371/journal.pone.0194883 (PMC5870983; doi:10.1371/journal.pone.0194883)
Supplement: S1 File — This document describes the consent that was signed by each patient included in this study. (PDF) [file pone.0194883.s001.pdf]

Ministère de la santé publique
Hôpital Charles Nicolle
Laboratoire d'immunologie
Pr. Yousr GORGI

Ministère de la santé publique
Hôpital Charles Nicolle
Service de rhumatologie
Pr. Leila Abdelmoula

Formulaire de consentement

Pour la participation à

L'étude immunogénétique et sérologique de l'IL-17 au cours de la polyarthrite rhumatoïde

De Mr/Mme ………………………………………………………………………………………… (nom, prénom)

Le Dr. .... m'a proposé(e) de participer à la recherche organisée par les Docteurs Yousr Gorgi et Leila Abdelmoula sur la polyarthrite rhumatoïde. Ce projet de recherche est intitulé : « étude immunogénétique et sérologique de l'IL-17 au cours de la polyarthrite rhumatoïde ».

Afin d'éclairer ma décision, j'ai reçu et bien compris les informations suivantes :

Je suis atteint(e) de polyarthrite rhumatoïde. Cette maladie multifactorielle possède une composante génétique multigénique complexe incluant l'implication du gène de l'IL-17. Cette cytokine serait une cible idéale chez certains malades pour améliorer le cours évolutif de la polyarthrite rhumatoïde. L'efficacité du traitement ciblant l'IL-17 dépend à la fois de facteurs génétiques et épigénétique influençant qualitativement et/ou quantitativement la production de cette cytokine.

La durée de ma participation à l'étude correspond à celle nécessaire à la réalisation d'un seul prélèvement sanguin (environ 10 ml).

Les analyses moléculaires visant à identifier l'impact des polymorphismes de l'IL-17 dans la survenue de cette maladie seront effectuées *in vitro* sur les acides nucléiques extraits des cellules sanguines.

Le dosage de l'IL-17 circulante sera réalisé sur le plasma recueilli à partir du même prélèvement.

Toutes les données recueillies et tous les résultats obtenus dans cette étude resteront strictement confidentiels et ne seront publiés, le cas échéant, dans des revues médicales que sous le sceau strict de l'anonymat. Je n'autorise leur consultation que par des personnes mandatées par les responsables de cette étude, organisateurs de la recherche, pour collaborer à celle-ci et, éventuellement, par un représentant des Autorités de Santé.

Je peux à tout moment demander toute information complémentaire auprès du Docteur ……………………………. (n°de tel. : ………………………….).

Après en avoir discuté et avoir obtenu réponse à toutes mes questions, j'accepte librement et volontairement de participer à la recherche décrite ci-dessus. Je suis parfaitement conscient que je peux retirer à tout moment mon consentement à ma participation à cette recherche et cela quelles que soient mes raisons et sans supporter aucune responsabilité. Le fait de ne plus participer à cette recherche ne portera pas atteinte à mes relations avec le médecin investigateur.

Le médecin m'a précisé que je suis libre d'accepter ou de refuser ; cela ne modifiera en rien, ni la prise en charge thérapeutique, ni les relations de confiance mutuelles établies entre moi-même et l'équipe soignante.

Mon consentement ne décharge en rien les organisateurs de la recherche de leurs responsabilités, et je conserve tous mes droits garantis par la loi.

Fait à ...., le ....

Signature

Signature de l'investigateur

Un exemplaire cosigné doit être remis à la personne qui participe à la recherche
